# Assessing the 53‐Year Epidemiological Trends of Oral Squamous Cell Carcinoma (OSCC) in Northeastern Iran

**DOI:** 10.1002/cre2.70092

**Published:** 2025-02-18

**Authors:** Nasrollah Saghravanian, Yasamin Tajdini, Pooya Saeedi, Mahsa Ghorbani

**Affiliations:** ^1^ Oral and Maxillofacial Disease Research Center, School of Dentistry Mashhad University of Medical Sciences Mashhad Iran; ^2^ School of Dentistry Mashhad University of Medical Sciences Mashhad Iran; ^3^ Dental Research Center, Mashhad University of Medical Sciences Mashhad Iran

**Keywords:** epidemiology, Iran, oral cancer, oral squamous cell carcinoma, prevalence

## Abstract

**Objectives:**

This study aimed to assess the epidemiological trends of oral squamous cell carcinoma (OSCC) in Northeast Iran over a 53‐year period.

**Material and Methods:**

In this retrospective study, we obtained data on OSCC cases in Northeast Iran spanning 53 years (1970–2022), including demographic details, such as age, sex, site, and histopathological grade. Descriptive analysis was conducted, and frequencies were compared using the chi‐squared test to evaluate differences between sexes, age groups, cancer sites, and histopathological grades. Additionally, we assessed the associations of each OSCC site with age, sex, and histopathological grade. Statistical significance was set at *p* < 0.05, with a confidence interval of 95%.

**Results:**

This study examined 13,769 cases, among which 507 were diagnosed with OSCC, accounting for 3.68% of the total cases. The mean age was 59.27 ± 14.94 years, with a significantly higher prevalence of OSCC among individuals older than 50 years (*p* < 0.001). There were nearly equal proportions of males and females, with the tongue being the most common site (28.4%), followed by the gingiva (26.4%) and buccal mucosa (19.5%). Grade 1 (well differentiated) OSCC was significantly more prevalent (54%) than other grades. There was a significant association between sex and tongue SCC in females (*p* = 0.006) and between sex and lip SCC in males (*p* = 0.008). Prevalence in individuals above 50 was significant for the tongue, buccal mucosa, gingiva, and lip (*p* = 0.001, < 0.001, < 0.001, 0.028, respectively). In the tongue, buccal mucosa, gingiva, and floor of the mouth, grade 1 was significantly more prevalent (*p* < 0.001).

**Conclusions:**

Our study revealed that OSCC predominantly affects individuals older than 50 years, with nearly equal prevalence between sexes. The most commonly affected sites are the tongue and gingiva, often exhibiting grade 1 histopathological findings. Enhanced community awareness of risk factors and regular oral examinations are essential for reducing the incidence of OSCC.

## 1 Introduction

Oral cancer ranks as the 16th most prevalent malignancy globally, with nearly two‐thirds of all new cases concentrated in Asian countries (Sung et al. 2021). Within the oral cavity, oral squamous cell carcinoma (OSCC) is the predominant malignant neoplasm, constituting more than 90% of all oral malignancies (Ling et al. 2021). It predominantly targets the tongue and floor of the mouth, with a greater prevalence in males than in females. Middle‐aged to elderly men are particularly susceptible, while its occurrence is notably less common among young adults (Deshmukh and Shekar 2021; Ferreira e Costa et al. 2022; Tan et al. 2023). Clinically, OSCC typically presents as ulcers, exophytic tumors, or patches of leukoplakia or erythroplakia (Minhas et al. 2019).

OSCC is a multifactorial disease influenced by genetic, environmental, and molecular factors. In the last decade, ferroptosis, a regulated cell death mechanism catalyzed by iron and mediated by peroxidation of polyunsaturated fatty acids, has emerged as a potential therapeutic strategy for multiple cancers, including OSCC (Zhou et al. 2024). Recent studies highlight its crucial role in OSCC development, tumor progression, and therapy resistance, making it an area of growing interest. Additionally, targeting the ferroptosis pathway has become a promising approach to treating advanced OSCC, warranting further research into its therapeutic potential (Kim et al. 2023).

The incidence of oral cancer exhibits geographical disparities worldwide. Lip and oral cavity cancers are notably prevalent in southern Asia and the Pacific Islands. Notably, in India and Sri Lanka, oral cancer is the foremost cause of cancer‐related mortality among men (Bai et al. 2020).

In Iran, cancer is the third leading cause of mortality (Saadat et al. 2015). According to the Globocan 2022 report, lip and oral cancer rank 21st and 22nd in terms of incidence and prevalence, respectively, among various cancers (Ferlay et al. 2024). The epidemiological characteristics of OSCC in Iran mirror patterns observed in other types of cancer (Maleki et al. 2015). Research conducted in academic centers and hospitals has revealed OSCC prevalence in major cities, such as Tehran, Mashhad, and Shiraz. The prevalence rates were approximately 1.29%–1.6% (Eshghyar et al. 2005; Sargeran et al. 2006), 0.9%–3.1% (Delavarian et al. 2009; Falaki et al. 2011; Jajarm and Ghodsi 2005), and 1.4%–5% (Andisheh‐Tadbir et al. 2008; Fahmy et al. 1983), respectively.

Epidemiological studies play a critical role in comprehending the prevalence, demographic features, and incidence of various cancers, as well as identifying specific risk factors. Moreover, these studies offer insights into the effectiveness of cancer control strategies. The valuable outcomes of such research guide the formulation and execution of cancer prevention and control initiatives (Alshami et al. 2023). In this context, our study aimed to collect demographic data and analyze clinical and pathological findings related to OSCC among patients at our medical center.

## 2 Methods

In this retrospective study, we conducted a comprehensive review of archives spanning from 1970 to 2022 originating from the Department of Oral and Maxillofacial Pathology, Mashhad University of Medical Sciences, Mashhad, Iran.

Our analysis involved a careful examination of 13,769 pathologic reports. We comprehensively identified and selected all oral and maxillofacial biopsies related to OSCC by employing a sampling census method. These selected cases underwent a validation process through microscopic reevaluation to ensure diagnostic accuracy and reliability.

Demographic information, including age, sex, and tumor location, was systematically collected for all cases of OSCC. Additionally, data on the histologic tumor grade, classified according to the WHO criteria (Barnes et al. 2005), were recorded. Cases lacking adequate demographic information or possessing unverified histopathological diagnoses were excluded to ensure the integrity and accuracy of our research. Sex was categorized as male or female, while age was computed annually and grouped into > 50 or ≤ 50 years. OSCC cases were classified based on their location, including Tongue, Lips, Gingiva, Palate, Buccal Mucosa, Floor of the Mouth, and Other. The histologic tumor grade was further classified as 1 (well differentiated), 2 (moderately differentiated), or 3 (poorly differentiated). A detailed analysis of tobacco and alcohol use was not conducted due to the unavailability of relevant data in the records.

This study is a retrospective analysis that utilized deidentified data from the database of Mashhad University of Medical Sciences. Since no identifiable patient information was accessed or used in the study, and the analysis did not involve direct contact with patients or intervention, the Ethics Committee of Mashhad University of Medical Sciences reviewed and waived the requirement for informed consent for this study.

### 2.1 Statistical Analysis

Our statistical analysis, conducted using SPSS software (version 27; IBM Corp., Armonk, NY, USA), included both descriptive and inferential components. Descriptive analysis offered insights through frequency and percentage distributions. To discern differences among sexes, age groups, OSCC sites, and different grades, inferential analysis initially employed appropriate tests. Further exploration involved assessing the correlation between each OSCC site and age, sex, and grade. The data were analyzed using the chi‐square test, the threshold for significance was set at *p* < 0.05, and the confidence intervals were set to 95%.

## 3 Results

According to our findings, out of the 13,769 cases examined, 507 were diagnosed with OSCC, accounting for approximately 3.68% of the total cases. The age distribution in our study ranged from 14 to 94 years, with a mean age of 59.27 ± 14.94 years. The prevalence of OSCC was significantly greater among individuals older than 50 years (*p* < 0.001). Notably, the seventh, eighth, and sixth decades had the highest prevalence of the disease, accounting for 140 cases (27.6%), 117 cases (23%), and 97 cases (19.1%), respectively. In contrast, the second and tenth decades had the lowest number of cases, with two and four cases, respectively.

**Figure 1:**
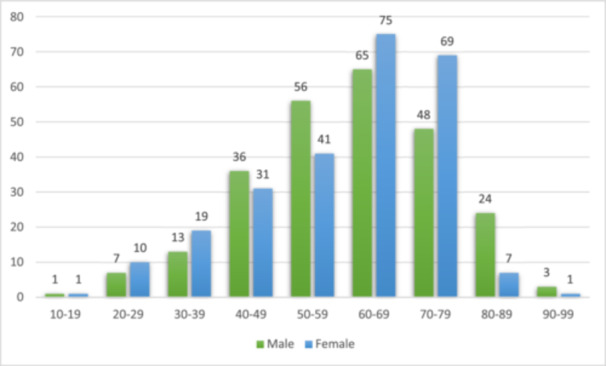
Distribution of all cases across decades and genders.

Among the total cases, 253 (49.9%) occurred in males and 254 (50.1%) occurred in females, reflecting an almost equal male‐to‐female ratio (0.99:1) and no significant difference (*p* = 0.970). However, in the fourth, seventh, and eighth decades, females exhibited a substantially greater prevalence than males, while in the sixth and ninth decades, males were more affected. Figure 1 illustrates the distribution of all cases across decades and sexes.

The most prevalent site for OSCC was the tongue, accounting for 144 cases (28.4%), followed by the gingiva in 134 cases (26.4%) and the buccal mucosa in 99 cases (19.5%). There were 29 cases involving the lip (5.7%), and 20 cases involving other sites in the mouth (3.9%) were the least affected.

Grade 1 OSCC was significantly more prevalent than other grades, constituting 274 cases (54%) (*p* < 0.001). Additionally, 189 cases (37.3%) were classified as Grade 2, and 44 cases (8.7%) were categorized as Grade 3. Table 1 describes the distributions of sex, age groups, tumor site, and pathological grade.

**Table 1:** Distribution of oral squamous cell carcinoma based on demographic variables (sex and age), site, and grade.

Variables	Frequency, %	*p*‐value
Sex		
Male	253, 49.9%	0.970
Female	254, 50.1%	
M:F^a^	0.99:1	
Age (years)		
≤ 50	148, 29.2%	<0.001*
> 50	359, 70.8%	
Mean age ± SD	59.27 ± 14.94	
Minimum–Maximum	14–94	
Site		
Buccal mucosa	99, 19.5%	<0.001*
Floor of the mouth	51, 10%	
Gingiva	134, 26.4%	
Lip	29, 5.7%	
Palate	30, 5.9%	
Tongue	144, 28.4%	
Other	20, 3.9%	
Grade		
1 (well differentiated)	274, 54.1%	<0.001*
2 (moderately differentiated)	189, 37.2%	
3 (poorly differentiated)	44, 8.7%	

^a^ Male to female ratio.

* Demonstrates a significant difference as a result of the *χ* ^2^ test, *p* < 0.05.

According to the chi‐square test, a noteworthy association emerged between sex and tongue SCC, highlighting a significant prevalence in females (*p* = 0.006). Additionally, a distinct connection was identified between sex and lip SCC, revealing a higher prevalence among males (*p* = 0.008). Conversely, sex differences at other sites were found to be statistically insignificant.

Furthermore, during the evaluation of age differences across oral sites, a significant prevalence was observed in individuals aged above 50 years for the tongue (*p* = 0.001), buccal mucosa (*p* < 0.001), gingiva (*p* < 0.001), and lip (*p* = 0.028).

Concerning the association between SCC grade and oral sites, our findings indicate that in the tongue, buccal mucosa, gingiva, and floor of the mouth, grade 1 is significantly more prevalent than other grades (*p* < 0.001 for all). However, other sites showed no significant difference in the prevalence of pathological grade. For details, please refer to Table 2.

**Table 2:** Correlations of oral sites with sex, age, and pathological grade.

	Sex (No., %)			Age, year (No., %)			Grade (No.)			
Site	Male	Female	*p*‐value	≤ 50	> 50	*p*‐value	1 (well differentiated)	2 (moderately differentiated)	3 (poorly differentiated)	*p*‐value
Tongue	55, 38%	89, 62%	0.006*	52, 36.1%	92, 63.9%	0.001*	77	57	10	< 0.001*
Buccal Mucosa	51, 52%	48, 48%	0.841	27, 27.3%	72, 72.7%	< 0.001*	61	29	9	< 0.001*
Palate	15, 50%	15, 50%	1.000	11, 36.7%	19, 63.3%	0.200	12	13	5	0.150
Gingiva	67, 50%	67, 50%	1.000	27, 20.2%	107, 79.8%	< 0.001*	74	49	11	< 0.001*
Lip	22, 76%	7, 24%	0.008*	8, 27.6%	21, 72.4%	0.028*	14	15	—	0.853
Floor of the Mouth	28, 55%	23, 45%	0.576	16, 31.3%	35, 68.7%	0.110	28	18	5	< 0.001*
Other	14, 70%	6, 30%	0.074	7, 35%	13, 65%	0.098	8	8	4	0.449

* Demonstrates a significant difference as a result of the chi‐squared test, *p* < 0.05.

## 4 Discussion

Oral cancer stands as a considerable global health concern, and its occurrence is intricately linked to sociocultural habits, resulting in notable variations in incidence across different regions (Curado et al. 2016). Epidemiological studies indicate an overall decline in its occurrence, accompanied by a surge in the prevalence of HPV‐positive oropharyngeal cancer in recent decades (Berman and Schiller 2017; Farquhar et al. 2018). Despite these trends, oral cancer remains the predominant malignancy among males in certain Asian countries (Abdulla et al. 2018).

According to the World Health Organization (WHO), in 2020, Iran reported 1139 new cases of oral cancer, with an incidence rate of 1.3 per 100,000 individuals, including 532 females and 607 males. Tobacco use among individuals aged 15 and above was prevalent at 14%, with 3.3% in women and 24.7% in men. Per capita alcohol consumption for those aged 15 and above averaged 1 liter per year, with 0.3 liters for women and 1.8 l for men (World Health Organization 2024).

The findings of the present study revealed an almost equal sex ratio of 0.99:1 between males and females, which contrasts with previous studies in Iran and other countries, which consistently reported a higher prevalence in males (Ferreira e Costa et al. 2022; Alshami et al. 2023; Ghafari et al. 2019). This discrepancy may be influenced by regional sociocultural factors. Historically, males have had higher rates of smoking and alcohol consumption, as well as increased occupational exposure to carcinogenic elements, such as ultraviolet radiation and human papillomavirus (HPV) (Alotaibi et al. 2019; Abbasi‐Kangevari et al. 2023; Reitsma et al. 2021; Nauta et al. 2021; Yang et al. 2023). However, recent trends indicate a decline in the male‐to‐female ratio of oral cancer cases globally, which could be attributed to increasing tobacco and alcohol consumption among women in recent decades (White 2020) Furthermore, cultural differences specific to the Khorasan Province, where our study was conducted, may have contributed to the observed near‐equal prevalence of OSCC between genders. Changes in smoking habits among women and improvements in healthcare access could also be factors influencing this trend.

In our study, the majority of OSCC patients were aged 50 and above, with the most affected age group being the seventh decade of life, consistent with findings from previous studies (Ferreira e Costa et al. 2022; Bai et al. 2020; Maleki et al. 2015; Alshami et al. 2023; Curado et al. 2016; Ghafari et al. 2019). Aging is recognized as a contributing factor to OSCC development and is influenced by a complex interplay of factors. Advanced age exposes individuals to heightened risk factors associated with OSCC (Tandon et al. 2017). Additionally, the decrease in natural defense mechanisms in the body with age increases the susceptibility of older individuals to cancer development (Duray et al. 2010). Moreover, the reduced capacity of the body to repair DNA damage as individuals age may further contribute to the initiation of OSCC (Gorbunova et al. 2007).

In this study, the tongue emerged as the predominant site for OSCC, consistent with findings from previous investigations in Iran and other countries (Ferreira e Costa et al. 2022; Bai et al. 2020; Maleki et al. 2015; Alshami et al. 2023; Ghafari et al. 2019). The increased incidence of tongue cancer can be ascribed to the proximity of the tongue to areas often exposed to carcinogens, such as tobacco and alcohol, thereby amplifying the risk of OSCC (Ferreira e Costa et al. 2022). Furthermore, persistent trauma in the form of prolonged irritation from orthodontic appliances, denture flanges, broken dental restorations, sharp teeth, and the positioning of the mandibular second molar may also be pivotal in the initiation and stimulation of neoplastic progression. Therefore, documenting the recent history of therapeutic procedures performed on patients is meaningful for understanding the potential contributing factors (Mohideen et al. 2019). Hence, considering the distribution of oral cancer across various sites, it appears impractical to concentrate solely on preventive and therapeutic strategies for one site. Instead, all sites with the potential for oral cancer should be taken into account. However, recognizing the limitations in resources, particularly within the healthcare system, and the necessity to prioritize therapy and prevention for this cancer, the suggestion is to prioritize the tongue due to its elevated rate of involvement.

Based on histological grades, our study revealed that Grade 1 OSCC was the most prevalent (54%), followed by Grade 2 (37.3%) and Grade 3 (2%). This pattern aligns with findings from Padma et al. (2017) and Akram et al. (2013), who also reported a higher percentage of grade 1 OSCC patients. However, these findings contrast with the results of Yasin et al. (2022), who reported a higher percentage of patients with grade 2 OSCC.

Our findings investigating the correlation between OSCC site and sex revealed a significantly higher prevalence of tongue OSCC in females and lip OSCC in males. This finding aligns with a study by Alshami et al. (2023)., which similarly revealed a significantly higher prevalence of lip OSCC in males, although other sites, including the tongue, showed no significant correlation with sex. Moreover, a comprehensive cohort study by Han et al. (2016), in the United States, exclusively assessing the prevalence of lip OSCC, reported a sex ratio of 4:1 between males and females, indicating a significantly greater prevalence of lip OSCC in males. However, the underlying reasons remain unknown. Future epidemiological studies focusing on social and environmental factors among lip SCC patients may provide further insights into the true relationship between sex and the predisposition to lip SCC. Regarding our finding of a significantly higher prevalence of tongue OSCC in females, recent discussions in the literature have highlighted an alarming increase in the incidence of base‐of‐the‐tongue OSCC in recent decades, particularly in women without traditional risk factors, such as alcohol or tobacco use (Kato et al. 2020). This trend is believed to be partially related to the substantial increase in the incidence of HPV‐associated oropharyngeal squamous cell carcinoma (Paderno et al. 2018). Ongoing investigations into additional genetic etiologic factors may contribute to explaining this evolving demographic profile of the disease.

Furthermore, our findings indicate a significant occurrence of OSCC in patients aged above 50 years, particularly in the tongue, buccal mucosa, gingiva, and lips. Conversely, other sites showed comparable occurrence across both age groups. However, in the study by Alshami et al. (2023), patients older than 40 years had a significantly greater incidence of OSCC in all locations. Regarding the association between histopathological grade and tumor site, all locations except for the lips and palate were found to have a significantly higher proportion of Grade 1 cases. Notably, this correlation has not been previously assessed in the literature.

Our findings align with several regional studies, providing additional context for the observed epidemiological trends. Razmpa et al. (2011) reported that the highest occurrence of tongue cancer was around the age of 60, which is consistent with our observation of a significant prevalence in the seventh and eighth decades. Similarly, Saghravanian et al. (2017) found that well‐differentiated squamous cell carcinoma (SCC) was the most frequent histopathological type, comprising 45.16% of SCC cases, which aligns with our finding that grade 1 SCC accounted for 54% of cases. Moreover, a systematic review by Maleki et al. (2015). highlighted the tongue as the most commonly affected site in oral cancer, with a prevalence of 30%, closely matching our result of 28.4%. However, a study by Ghafari et al. (2019). reported a male‐to‐female ratio of 1.48:1 among OSCC cases, which contrasts with our nearly equal ratio of 1:1. Despite this difference, their finding that the tongue was the most common site of OSCC (38.2%) is in agreement with our results. These comparisons underscore the relevance of regional data in contextualizing our findings and highlight both consistencies and disparities in the epidemiology of OSCC.

In recent years, emerging research has highlighted the potential role of ferroptosis in the pathogenesis of OSCC. Ferroptosis, a regulated form of cell death induced by iron‐dependent lipid peroxidation, has shown promising therapeutic potential in treating various cancers, including OSCC. This process leads to cell membrane rupture and tumor suppression (Zhou et al. 2024; Kim et al. 2023). The role of ferroptosis in OSCC development has gained considerable attention, with increasing evidence suggesting its influence on tumor growth, metastasis, and therapy resistance. Furthermore, oxidative stress, a key player in OSCC pathogenesis, interacts with ferroptosis to promote cancer progression (Zhou et al. 2024). In addition to ferroptosis, other risk factors, such as HPV infection, tobacco, and alcohol use, continue to be important contributors to OSCC development (Nokovitch et al. 2023). The interplay of these factors with emerging molecular mechanisms offers new insights into potential therapeutic targets and highlights the need for further investigation into their roles in OSCC.

The strengths of our study include the large number of cases evaluated and the examination of different aspects of the disease. Additionally, our study stands out as one of the few to assess cases over a 53‐year period, which should be regarded as a notable feature. Despite our efforts to consider risk factors and habits, such as tobacco and alcohol consumption, as well as other variables such as prognosis, we omitted data from our analysis due to a lack of information and a significant number of cases with missing data, which could potentially limit our study. Future research should focus on investigating these risk factors and analyzing correlations in different populations to better understand geographical variations in risk factors and disease behaviors.

## 5 Conclusion

In summary, our 53‐year study in Northeast Iran revealed that OSCC predominantly affects individuals older than 50 years, with a near‐equal prevalence between the sexes. The tongue and gingiva are the most commonly affected sites, with Grade 1 being the predominant histopathological grade. To reduce the incidence of OSCC, enhancing community awareness of risk factors is vital, as is regular oral examinations for early detection.

## Author Contributions

**Nasrollah Saghravanian:** conceptualization, methodology, validation, supervision, project administration, writing – review and editing. **Yasamin Tajdini:** investigation, conceptualization, writing – review and editing. **Pooya Saeedi:** conceptualization, methodology, investigation, writing – original draft, writing – review and editing. **Mahsa Ghorbani:** investigation, methodology, visualization, writing – original draft, writing – review and editing.

## Ethics Statement

The Ethics Committee of Mashhad University of Medical Sciences granted approval for this study, and permission was secured to access the database utilized. Following data collection, all information was deidentified before analysis.

## Consent

The authors have nothing to report.

## Conflicts of Interest

The authors declare no conflicts of interest.

## Data Availability

The data that support the findings of this study are available from the corresponding author upon reasonable request.
